# Comparative modulation of lncRNAs in wild-type and *rag1*-heterozygous mutant zebrafish exposed to immune challenge with spring viraemia of carp virus (SVCV)

**DOI:** 10.1038/s41598-019-50766-0

**Published:** 2019-10-02

**Authors:** Valentina Valenzuela-Muñoz, Patricia Pereiro, Margarita Álvarez-Rodríguez, Cristian Gallardo-Escárate, Antonio Figueras, Beatriz Novoa

**Affiliations:** 1 Laboratory of Biotechnology and Aquatic Genomics, Interdisciplinary Center for Aquaculture Research (INCAR), University of Concepción, Concepción, Chile; 2 Instituto de Investigaciones Marinas (IIM), CSIC, Vigo, Spain

**Keywords:** Immunogenetics, Genomics

## Abstract

Although the modulation of immune-related genes after viral infection has been widely described in vertebrates, the potential implications of non-coding RNAs (ncRNAs), especially long non-coding RNAs (lncRNAs), in immunity are still a nascent research field. The model species zebrafish could serve as a useful organism for studying the functionality of lncRNAs due to the numerous advantages of this teleost, including the existence of numerous mutant lines. In this work, we conducted a whole-transcriptome analysis of wild-type (WT) and heterozygous *rag1* mutant (*rag1*^+/−^) zebrafish after infection with the pathogen spring viraemia of carp virus (SVCV). WT and *rag1*^+/−^ zebrafish were infected with SVCV for 24 h. Kidney samples were sampled from infected and uninfected fish for transcriptome sequencing. From a total of 198,540 contigs, 12,165 putative lncRNAs were identified in zebrafish. Most of the putative lncRNAs were shared by the two zebrafish lines. However, by comparing the lncRNA profiles induced after SVCV infection in WT and *rag1*^+/−^ fish, most of the lncRNAs that were significantly induced after viral challenge were exclusive to each line, reflecting a highly differential response to the virus. Analysis of the neighboring genes of lncRNAs that were exclusively modulated in WT revealed high representation of metabolism-related terms, whereas those from *rag1*^+/−^ fish showed enrichment in terms related to the adaptive immune response, among others. On the other hand, genes involved in numerous antiviral processes surrounded commonly modulated lncRNAs, as expected. These results clearly indicate that after SVCV infection in zebrafish, the expression of an array of lncRNAs with functions in different aspects of immunity is induced.

## Introduction

The increasing genomic data available for model and non-model organisms has revealed that only a small percentage of the genome corresponds to protein-coding genes. A high proportion of the genome does not present coding potential but is transcribed into non-coding RNAs (ncRNAs). Among ncRNAs, the long non-coding RNAs (lncRNAs) present a length of over 200 nucleotides (nt) and are transcribed in the same way as mRNAs. LncRNAs regulate the expression of adjacent or distal genes through different mechanisms, such as transcription interference, initiation of chromatin remodeling, promoter inactivation, activation of accessory proteins, and activation of transcription factors^1^. Because most lncRNAs influence the expression of their neighbor genes by acting as local regulators, lncRNA expression is often positively or inversely correlated with the expression of their adjacent coding-genes^1^. Notably, lncRNAs play a role in different molecular processes and exhibit specific-transcription patterns in different cell types^2^, where these transcripts can promote or repress the gene expression^3^. Most likely due to the functional complexity of lncRNAs, studies concerning their activity remain scarce.

For understanding the role of lncRNAs, one of most useful species is zebrafish (*Danio rerio*). Zebrafish is a model fish species for genomic, developmental, biomedical and pharmacological studies, among other areas of inquiry. Studies on lncRNAs in zebrafish have demonstrated their roles in processes such as tissue and organ repair^3^, development and nervous system function^4^, cold acclimation^5^, the response to antibiotics^6^, and immune system function^7^.

In the case of the fish immunity, lncRNAs are key players that in turn can display complex mechanism to modulate the expression of Toll-like receptors, NF-kB activity, differentiation of the Th1/Th2 response, and other immune-relevant functions^2^. For instance, in mammals, the lncRNA Morrbid, HOTAIRM1 or lnc-DC mediates the development and differentiation of dendritic and myeloid cells^8^. Nevertheless, studies focused on the activity of lncRNAs in the teleost immune system are still scarce. In zebrafish, knockdown analysis demonstrated the role of the PU.1 gene in adaptive immunity and the negative regulation of this gene by its antisense long non-coding RNA (AS lncRNA)^7^. Furthermore, different expression patterns of lncRNAs have been described in response to a variety of pathogens in salmonids^9–12^, and also the modulation of the lncRNA patterns by HSP70 and *Streptococcus agalactiae* antigen stimulation in tilapia (*Oreochromis niloticus*) has been recently analyzed^13^.

Moreover, an excellent model for understanding the role of lncRNAs in the immune response and their modulation after infections is the zebrafish mutant line *rag1*. V(D)J recombination, carried out by the combined endonuclease activity of recombination activating gene 1 (RAG1) and RAG2, assembles the vast diversity of immunoglobulins and T cell receptor (TCR) genes^14^. A point mutation of the *rag1* gene in zebrafish causes a premature stop codon in the *rag1* catalytic domain; therefore, this mutation presumably abolishes the adaptive immune system^15^. Herein, SVCV is an enveloped, negative-sense, single-stranded RNA virus belonging to the *Rhabdoviridae* family, and this pathogen mainly affects cyprinids, including zebrafish^16,17^. Although numerous investigations concerning the immune system have been developed in zebrafish using SVCV^18–21^, to our knowledge, this is the first time that the impact of this virus on the lncRNA profile has been analyzed.

Comparison of the lncRNA expression pattern after SVCV challenge in wild-type (WT) and *rag1*-heterozygous mutant fish (*rag1*^+/−^) would allow us to elucidate the potential implications of the diversity of lncRNAs in different aspects of innate and adaptive immunity. Because heterozygous *rag1* mutants are partially deficient in the generation of mature lymphocytes, the potential compensation mechanisms induced after SVCV challenge could reveal specific lncRNAs related to acquired immunity. This study gives new genomic knowledge of how lncRNAs are key molecular components of the immune system in teleost.

## Methods

### Zebrafish and virus

Six-month-old WT and *rag1*^+/−^ zebrafish were obtained from the facilities of the Instituto de Investigaciones Marinas (Vigo, Spain), where zebrafish are maintained following established protocols^22,23^. Zebrafish were euthanized using a tricaine methanesulfonate (MS-222) overdose (500 mg/l). Fish care and challenge experiments were conducted according to the guidelines of the CSIC National Committee on Bioethics under approval number ES360570202001/16/FUN01/PAT.05/tipoE/BNG.

Spring viraemia of carp virus (SVCV) isolate 56/70 was propagated on epithelioma papulosum cyprini (EPC) carp cells (ATCC CRL-2872) containing MEM (Gibco) supplemented with 2% FBS (Gibco) and 100 µg/ml Primocin (InvivoGen) and was titrated in 96-well plates. The TCID_50_/ml was calculated according to the Reed and Muench method^24^.

### Experimental design and samples

Twelve zebrafish from each line (WT and *rag1*^+/−^) were intraperitoneally (i.p.) injected with 20 μl of SVCV (3 × 10^2^ TCID_50_/ml), and as control groups, the same number of fish were injected with an equal volume of MEM (Gibco, USA) supplemented with 2% FBS (Gibco) and 100 µg/ml Primocin (InvivoGen, USA). Kidney samples were collected at 24 h post-injection (hpi) and stored at −80 °C until RNA extraction.

In parallel, the effect of the virus on the survival of heterozygous mutants was evaluated. For this purpose, 20 individuals from each line (WT and *rag1*^+/−^) were also i.p. infected with the virus, and another 20 fish were inoculated with the medium. The 20 zebrafish from each group were divided into 2 batches of 10 fish/batch, which served as biological replicates for the survival analysis. Mortality was recorded over a period of 3 weeks. Kaplan-Meier survival curves were analyzed with a log-Rank (Mantel-Cox) test.

### High-throughput transcriptome sequencing

Kidney samples from infected and control WT and *rag1*^+/−^ zebrafish were used for Illumina cDNA library preparations. Briefly, total RNA was extracted from each individual using the Maxwell^®^ 16 LEV simplyRNA Tissue kit (Promega) with an automated Maxwell 16 Instrument following the manufacturer’s instructions. The quantity and purity of the total RNA was evaluated by using a Nanodrop ND-1000. RNA integrity was analyzed using the Bioanalyzer 2100 system (Agilent Technologies Inc., USA) according to the manufacturer’s instructions. Samples with a RIN over 8.0 were pooled and used for library preparation. Subsequently, using the RNA pools from the control and challenged groups (3 biological replicates; 4 fish/replicate), double-stranded cDNA libraries were constructed using the TruSeq RNA Sample Preparation Kit v2 (Illumina^®^, San Diego, CA, USA). Samples were sequenced by using the HiSeq (Illumina^®^) platform. The raw read sequences were deposited in the Sequence Read Archive (SRA) (https://www.ncbi.nlm.nih.gov/sra) under accession number PRJNA532380.

### Sequence assembly and lncRNA identification

Sequence assembly was carried out using CLC Genomics Workbench v10.1 software (CLC Bio, Aarhus, Denmark). *De novo* assembly was performed using datasets from the zebrafish group. Assembly was conducted with an overlap criterion of 70% and a similarity of 0.9 to exclude paralogous sequence variants (PSVs)^25^. The settings were as follows: a mismatch cost of 2, deletion cost of 3, insert cost of 3, minimum contig length of 200 base pairs (bp) and trimming quality score of 0.05. After the assembly process, singletons were retained in the dataset as possible representatives of low-expression transcript fragments. However, the sequence redundancy of these fragments was removed by using the Duplicate Finder application incorporated in Geneious v8.0 software (Biomatters, Auckland, New Zealand). The assembled data were processed using CLC Genomics Workbench software following the previously described pipeline^10,11^. Briefly, following *de novo* assembly of the WT and *rag1*^+/−^ zebrafish transcriptomes, several filters were applied to the consensus sequences of contigs. Sequences with an average coverage <50 were discarded. Then, BlastX analysis was performed for selected sequences, discarding all sequences with positive Blast hits (BlastX, e-value < 1e-05) against the proteins for all the bony fish species included in the NCBI GenBank and UniProt databases. Open reading frames (ORFs) were predicted from the remaining contigs, and all contigs with putative ORFs > 200 bp were discarded. Finally, the free Coding Potential Assessment Tool (CPAT) (http://lilab.research.bcm.edu/cpat/) was used to discard sequences with coding potential.

### Genome mapping and pathway analysis

To gain a better understanding of the genomic context of lncRNAs in WT and *rag*^+/−^ zebrafish, non-coding transcripts were mapped against the last version of the zebrafish genome (Assembly: GCA_000002035.4 GRCz11). Thus, lncRNAs were mapped using the CLC Genomics Workbench mapping algorithm and considering the following parameters: length fraction = 0.8, similarity fraction = 0.8 and mismatch, insertion and deletion costs of 2, 3 and 3, respectively. In addition, coding genes flanking up to 10,000 bases upstream and downstream from the annotated lncRNAs were identified and extracted for GO and KEGG analysis. Thus, GO enrichment analysis^26^ was conducted to identify the most represented biological process, cellular component and molecular function categories among the protein coding genes proximal to lncRNAs. The results were finally plotted using the REVIGO platform^27^, R and CYTOSCAPE software^28^.

### RNA-Seq analysis

The RNA-Seq settings included a minimum length fraction = 0.6 and a minimum similarity fraction (long reads) = 0.5. The expression values were set to the transcripts per million model (TPM). The distance metric was calculated using the Manhattan method, with the mean expression level in 5–6 rounds of k-means clustering subtracted. Finally, Kal’s statistical analysis test was used to compare gene expression levels in terms of the log2 fold-change (P = 0.0005; FDR corrected).

### Quantitative PCR (qPCR) validation of lncRNA expression

cDNA synthesis from the samples was conducted with the NZY First-Strand cDNA Synthesis kit (NZYTech) using 0.5 μg of total RNA. A total of 12 lncRNAs were used to validate the RNA-Seq results. Specific qPCR primers for the selected lncRNAs were designed using Primer 3 software^29^, and their amplification efficiency was calculated with the threshold cycle (CT) slope method^30^. The primer sequences used for qPCR are listed in Supplementary Table S1. Individual qPCR assays were carried out in a 25 μl reaction volume containing 12.5 μl of SYBR GREEN PCR Master Mix (Applied Biosystems), 10.5 μl of ultrapure water, 0.5 μl of each specific primer (10 μM) and 1 μl of two-fold-diluted cDNA template in MicroAmp optical 96-well reaction plates (Applied Biosystems, USA). Reactions were conducted using technical triplicates in a 7300 Real-Time PCR System thermocycler (Applied Biosystems). The qPCR conditions were as follows: initial denaturation step (95 °C, 10 min), 40 cycles of a denaturation step (95 °C, 15 s) and a hybridization-elongation step (60 °C, 1 min). The relative expression level of the lncRNAs was normalized following the Pfaffl method^30^ and using the *18S ribosomal RNA* (*18 s*) as a reference gene.

## Results

### Survival rate of WT and rag1^+/−^ zebrafish after SVCV challenge

In contrast to what is observed in homozygous *rag1* mutant zebrafish, which are more resistant to infection with SVCV compared to WT fish^31,32^, no significant differences in survival were found between the WT and *rag1*-heterozygous fish after SVCV challenge (Supplementary Fig. S1).

### Sequence assembly and lncRNA identification

**Figure 1:**
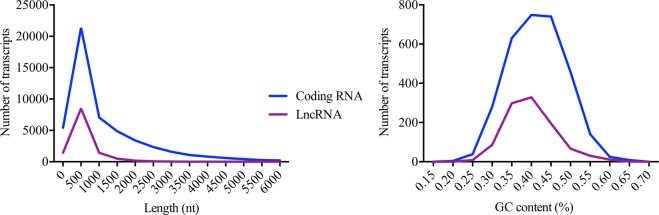
Features of predicted lncRNAs from the WT and *rag1*^+/−^ zebrafish transcriptomes. (**A**) Length distribution of predicted lncRNAs and coding transcripts. (**B**) Comparison of GC content between lncRNAs and coding transcripts.

**Figure 2:**
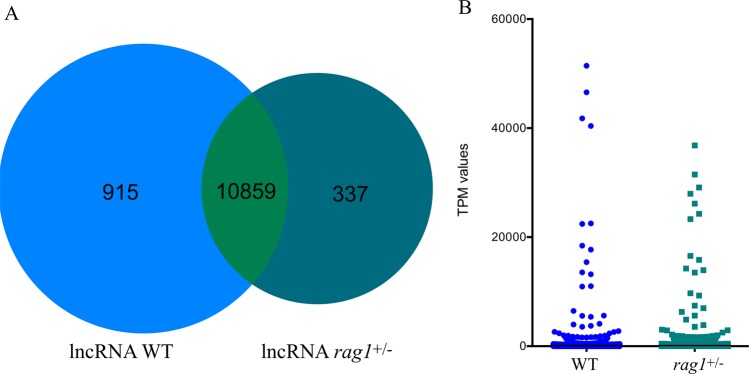
Features of predicted lncRNAs expressed in kidneys of WT and *rag1*^+/−^ zebrafish. (**A**) Venn diagram representation of shared and exclusive lncRNAs constitutively expressed in both zebrafish lines. (**B**) Profile of TPM values for common lncRNAs expressed in WT and *rag1*^+/−^ zebrafish.

**Figure 3:**
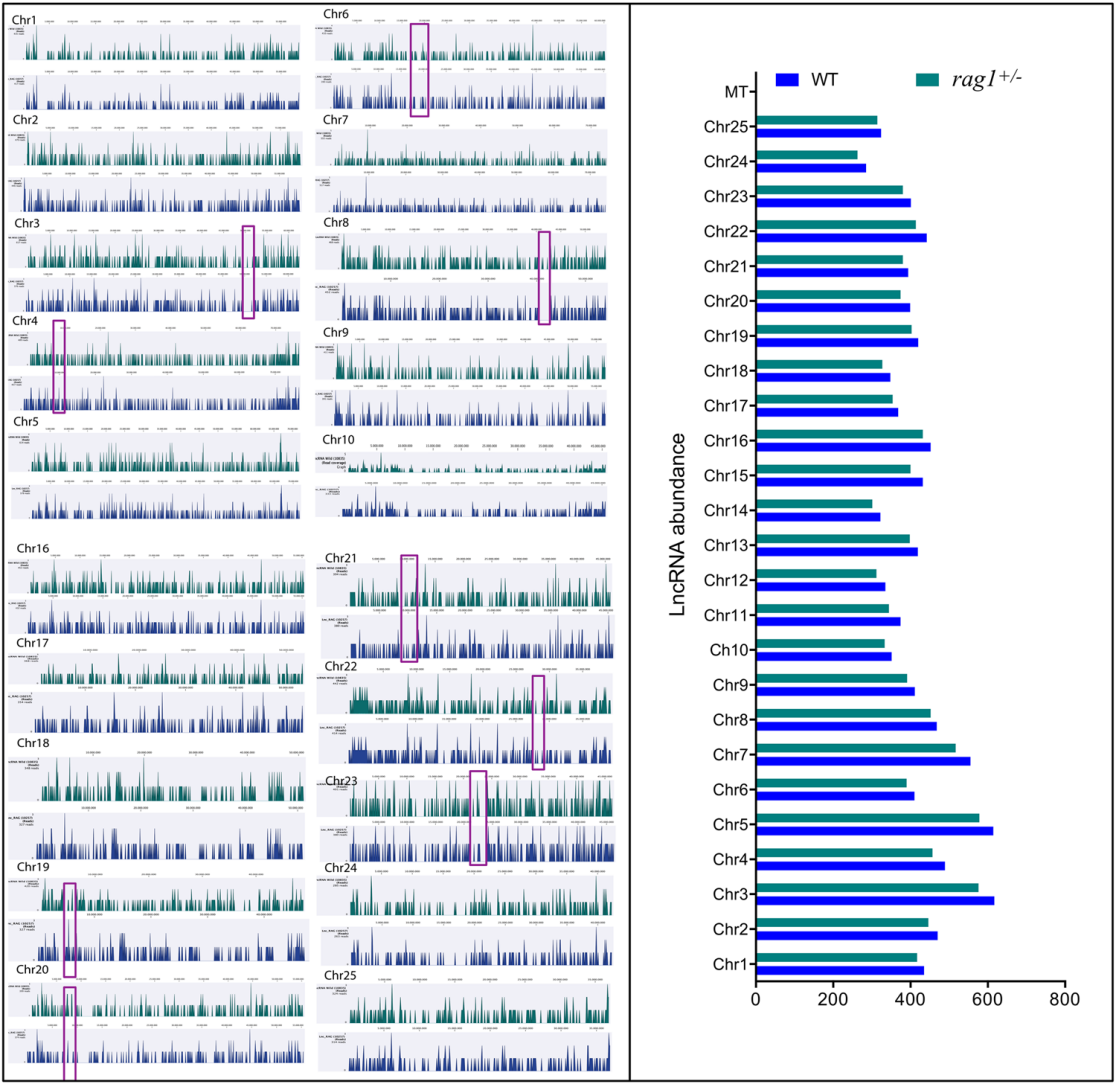
LncRNA abundance and chromosome localization in WT and *rag1*^+/−^ zebrafish. LncRNAs were mapped in the zebrafish genome to identify potential differences in the chromosome location between WT and *rag1*^+/−^ zebrafish. In general terms, lncRNAs from both lines showed a similar chromosome distribution and abundance per chromosome. Nevertheless, some subtle differences were observed, revealing the specific position of WT and *rag1*^+/−^ exclusive lncRNAs.

A total of 543,596,316 and 651,786,032 reads were obtained for WT and *rag1*^+/−^ zebrafish, respectively. From the total assembly, 198,540 contigs were obtained from WT and *rag1*^+/−^ zebrafish samples, among which 58,805 contigs represented coding sequences, and 12,165 were putative lncRNA sequences, with average lengths of 832 nt and 545 nt, respectively. The lncRNAs identified in zebrafish presented a length distribution ranging from 200 to 2,000 nt, whereas for the coding sequences, this range was 200 to 5,500 nt (Fig. 1). Moreover, 11,774 and 11,196 lncRNAs were identified from each dataset for WT and *rag1*^+/−^ zebrafish, respectively, with the two strains sharing 10,859 lncRNAs (Fig. 2A) with similar expression profiles (Fig. 2B). Additionally, the locations of the lncRNAs identified for WT and *rag1*^+/−^ zebrafish in the zebrafish genome were determined. Although few differences in lncRNA localization were observed for the two zebrafish lines, *rag1*^+/−^ zebrafish did present some lncRNAs in different positions than the WT fish (Fig. 3).

**Figure 4:**
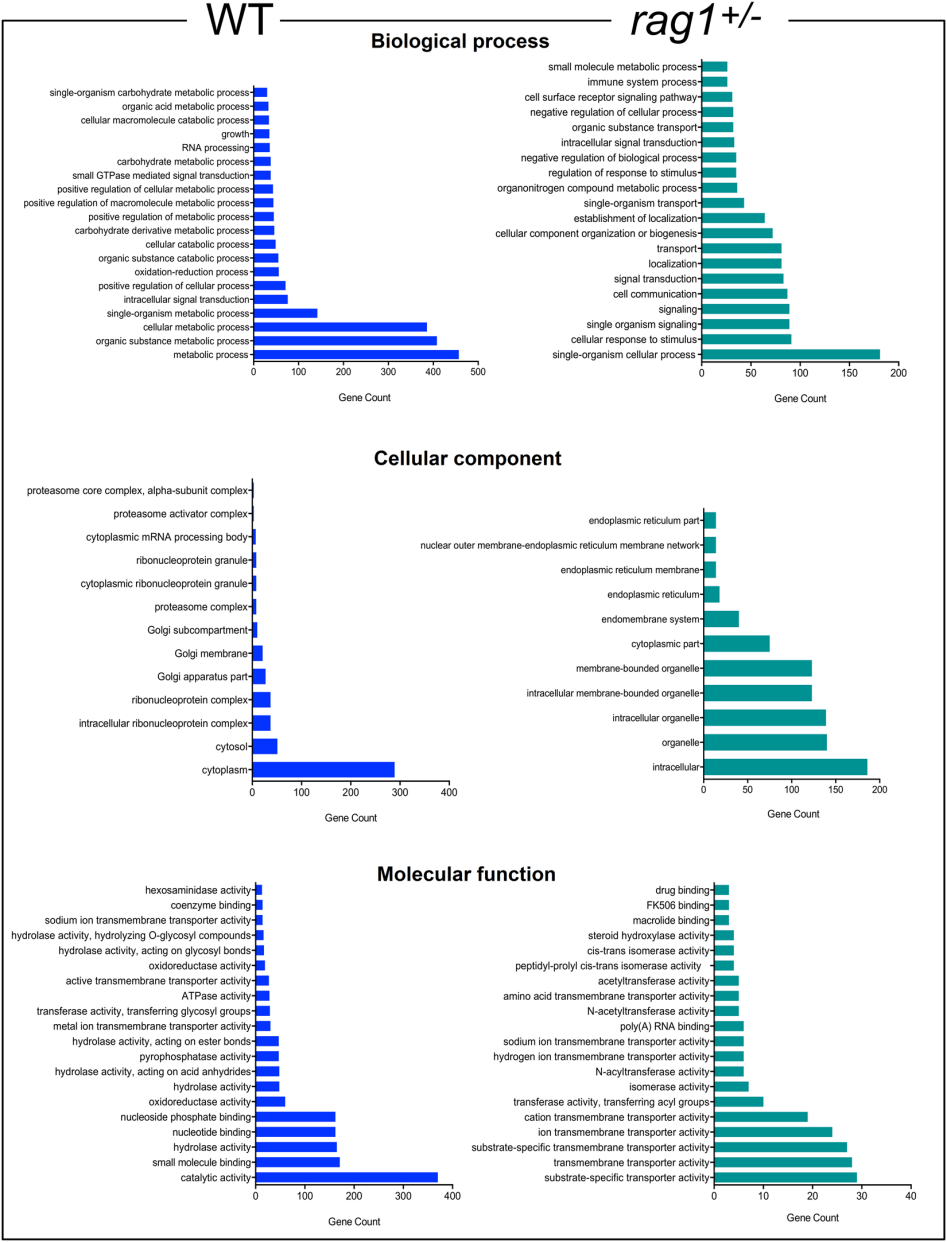
GO analysis of exclusive lncRNA-neighboring genes identified in WT and *rag1*^+/−^ zebrafish. Those lncRNAs exclusively detected in WT or *rag1*^+/−^ fish were positioned on the genome and the protein-coding genes surrounding the lncRNAs were assigned to biological process, cellular component and molecular function categories.

The neighboring genes of the exclusive lncRNAs of WT and *rag1*^+/−^ zebrafish were annotated by GO analysis, showing different processes potentially modulated by these exclusive lncRNAs (Fig. 4). The biological processes that were putatively regulated by exclusive lncRNAs of WT zebrafish were mainly associated with different metabolic processes; for the cellular components, the greatest number of lncRNA-neighboring genes were associated with cytoplasm; and catalytic activity was the molecular function with the most 7-annotated genes (Fig. 4). In contrast, the exclusive lncRNAs of *rag1*^+/−^ zebrafish were located near genes annotated to biological processes related to the cellular response to stimulus, cell communication, transport and immune response categories, among other processes. Among cellular components, intracellular and organelle components were the most abundant components annotated, and in the molecular function category, the most abundant neighboring genes were annotated as being associated with substrate-specific transporter activity and transmembrane transporter activity (Fig. 4).

### Neighboring lncRNAs to rag1 and rag2 genes chromosomal localization and modulation

**Figure 5:**
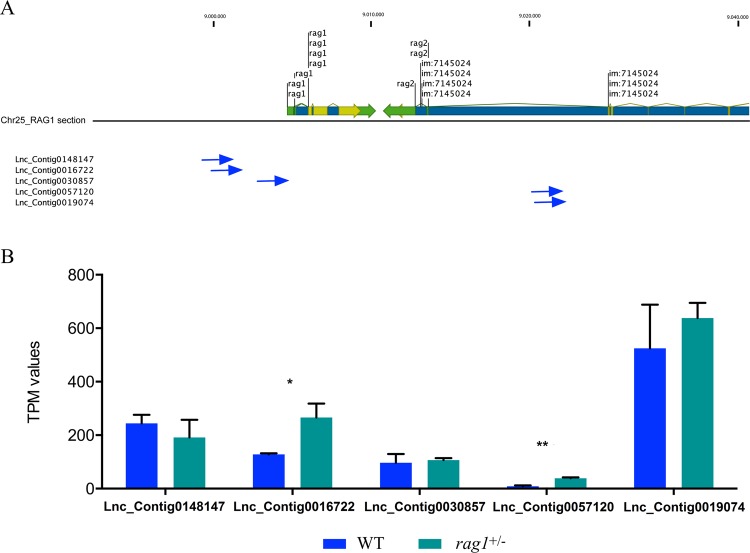
Chromosomal location and constitutive expression of the *rag1* and *rag2* neighboring lncRNAs. (**A**) LncRNA mapping in chr25 near to *rag* genes. (**B**) Expression profile of *rag1* and *rag2* neighboring lncRNAs. (**p value < 0.005, *p value < 0.5).

Five lncRNAs identified in WT and *rag1*^+/−^ zebrafish showed a physical linkage with *rag1* and *rag2* genes on chromosome 25 (Fig. 5A). Expression analysis of these lncRNAs using the TPM values of the samples revealed that two of them were differentially expressed between WT and *rag1*^+/−^ zebrafish (Fig. 5B). The Lnc_Contig0016722 and Lnc_Contig0057120 were up-regulated in *rag1*^+/−^ compared to the WT zebrafish. The results suggest a putative regulatory role of Lnc_Contig0016722 and Lnc_Contig0057120 in the expression of *rag1* and *rag2* genes in *rag1*^+/−^ zebrafish.

### LncRNA modulation during SVCV infection

**Figure 6:**
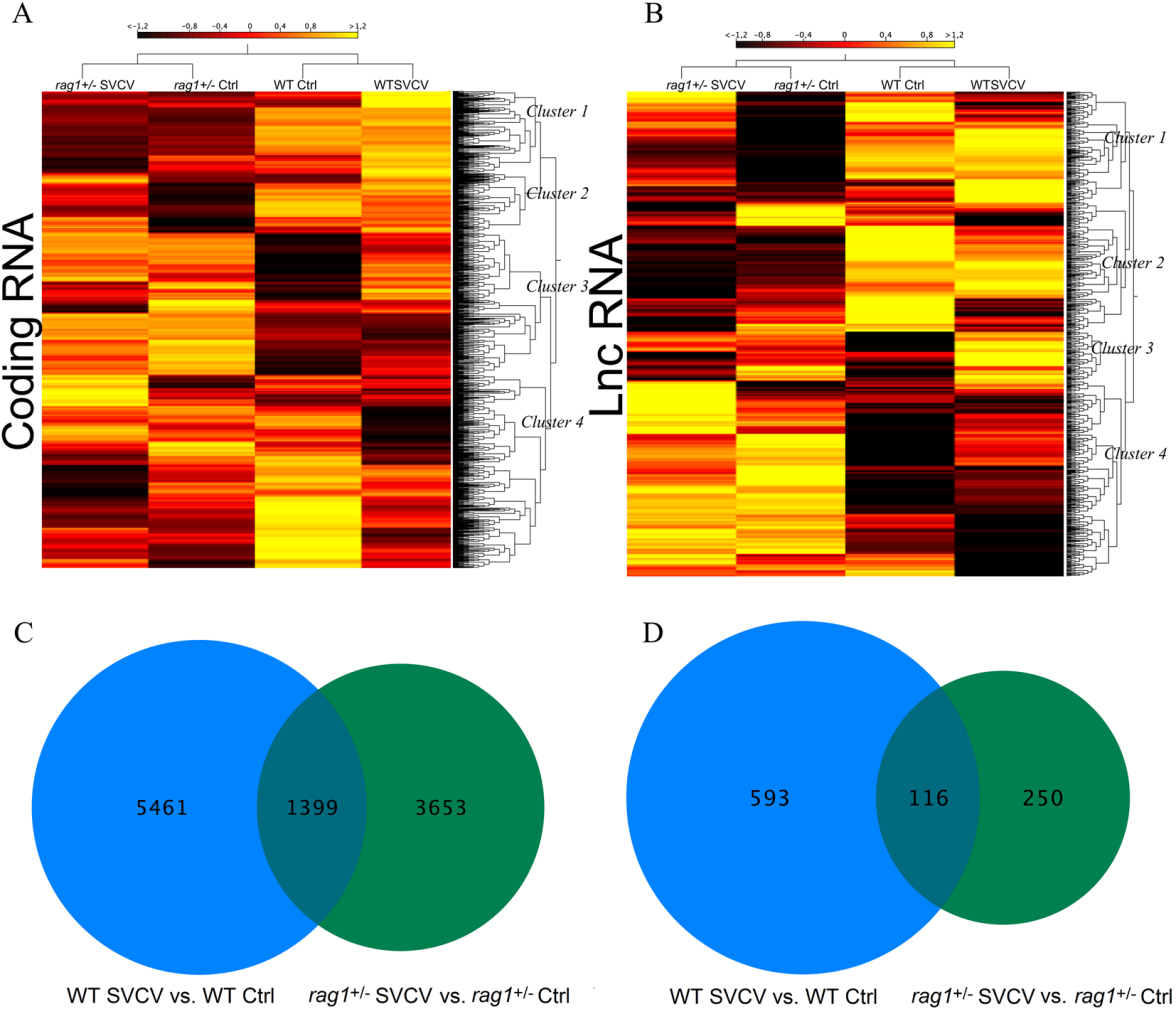
Differentially expressed transcripts in response to SVCV infection. (**A**,**B**) Hierarchical clustering of expressed transcripts (TPM values) in kidney samples from different experimental conditions. (**A**) Coding transcripts. (**B**) LncRNAs. (**C**,**D**) Venn diagrams representing the significantly expressed, (**C**) coding transcripts and (**D**) lncRNAs (fold-change 4, p-value 0.01).

RNA-Seq analysis of coding and non-coding transcripts in the zebrafish samples revealed two differentiated clusters of samples, one for control and SVCV-infected WT zebrafish and another cluster for both conditions in *rag1*^+/−^ zebrafish, reflecting greater relevance of the zebrafish line than the infection (Fig. 6A,B). The heat map representation of protein-coding transcripts exhibited four clusters of contigs with a similar expression profile (Fig. 6A). Cluster 1 was highly expressed in both control and infected WT samples; cluster 2 showed greater representation in control and infected *rag1*^+/−^ zebrafish and infected WT; cluster 3 was mainly expressed in control and infected *rag1*^+/−^ zebrafish; and cluster 4 was mainly expressed in control WT fish. The protein-coding contigs included in each cluster are represented in Supplementary Table S2. Moreover, four main lncRNA clusters were identified from the heat map representation with similar expression profiles to those observed for the coding transcripts. The first (cluster 1) included those lncRNAs that were downregulated in *rag1*^+/−^ control zebrafish (Fig. 6B), suggesting a role of these lncRNAs in the response to SVCV infection in *rag1*^+/−^ zebrafish. On the other hand, cluster 2 was mainly up-regulated in kidney samples from control and infected WT zebrafish (Fig. 6B). Cluster 3 was highly regulated in control *rag1*^+/−^ zebrafish and infected WT individuals. Finally, cluster 4 was more highly expressed in control and infected *rag1*^+/−^ zebrafish (Fig. 6B). The lncRNAs included in the different clusters are represented in Supplementary Table S2.

In addition, the differential expression analysis (absolute fold-change of 4 and p-value of 0.001) between SVCV-challenged and control WT and *rag1*^+/−^ zebrafish showed a greater number of protein-coding and lncRNA transcripts that were modulated during infection in WT zebrafish (6,860 and 709, respectively) than in *rag1*^+/−^ zebrafish (5,052 and 366). Interestingly, Venn diagrams showed that the majority of these transcripts were exclusively modulated after infection in each line, with 5,461 coding RNAs and 593 lncRNAs being exclusively modulated in WT and 3,653 coding RNAs and 250 lncRNAs being exclusively modulated in *rag1*^+/−^ zebrafish (Fig. 6C,D). Moreover, a total of 116 common coding-genes and 1,399 common lncRNAs were identified between WT and *rag1*^+/−^ zebrafish. Supplementary Table S3 shows the most modulated common and exclusive lncRNAs in WT and *rag1*^+/−^ after infection, reflecting that some commonly modulated lncRNAs were regulated in opposite ways.

### Enrichment of the neighboring genes of lncRNAs expressed during infection

**Figure 7:**
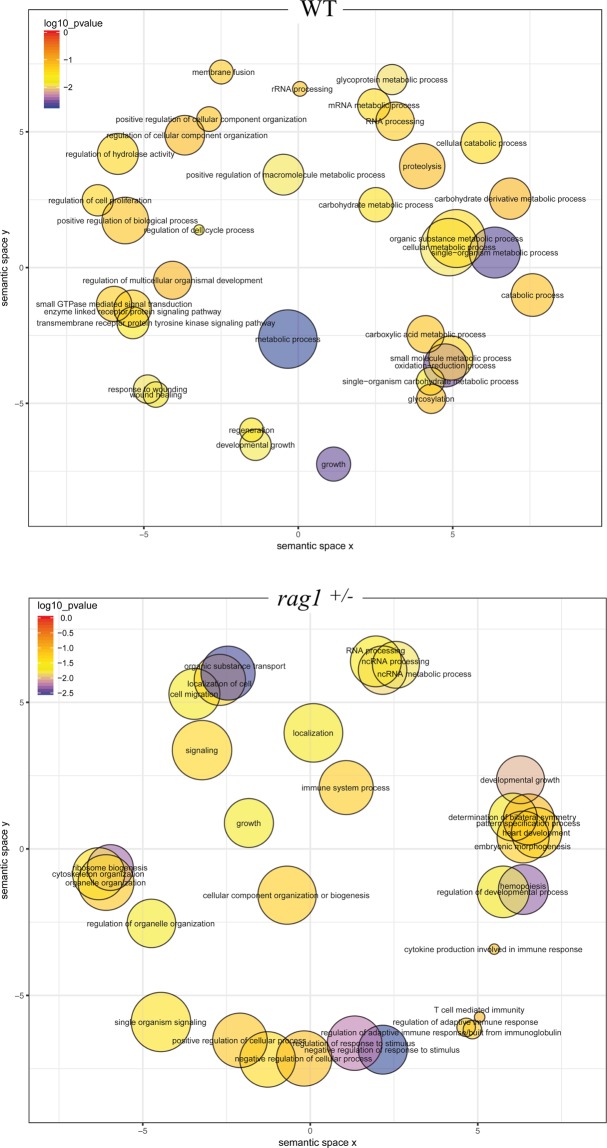
GO enrichment analysis for exclusive lncRNA-neighboring genes annotated in WT and *rag1*^+/−^ zebrafish that were highly regulated during viral infection. Although most of the lncRNAs modulated after an SVCV challenge were common to both zebrafish lines, some lncRNAs were exclusively regulated in WT and *rag1*^+/−^ fish. The protein-coding genes located within 10 kb upstream and downstream from these lncRNAs were assigned to GO terms and a GO enrichment analysis was conducted.

Exclusive lncRNAs expressed after infection in WT and *rag1*^+/−^ zebrafish were located in the genome to identify neighboring protein-coding genes located within 10 kb upstream and downstream from the lncRNAs (Supplementary Table S4). For the WT zebrafish-exclusive lncRNAs, GO enrichment of neighboring genes revealed that the most represented biological processes were different metabolic processes, growth, and oxidation-reduction process, among others (Fig. 7A). Interestingly, for the *rag1*^+/−^ zebrafish-exclusive lncRNA-neighboring genes, several biological processes related to immunity, especially to adaptive immunity, were observed (immune system process, T cell-mediated immunity, regulation of adaptive immune response/built from immunoglobulin, regulation of adaptive immune response, cytokine production involved in immune response) (Fig. 7B). Similar results were observed for the GO enrichment analysis of exclusive coding RNAs expressed differentially during infection. A greater number of *rag1*^+/−^ exclusive coding RNAs were associated with the immune response process (Supplementary Fig. S2).

**Figure 8:**
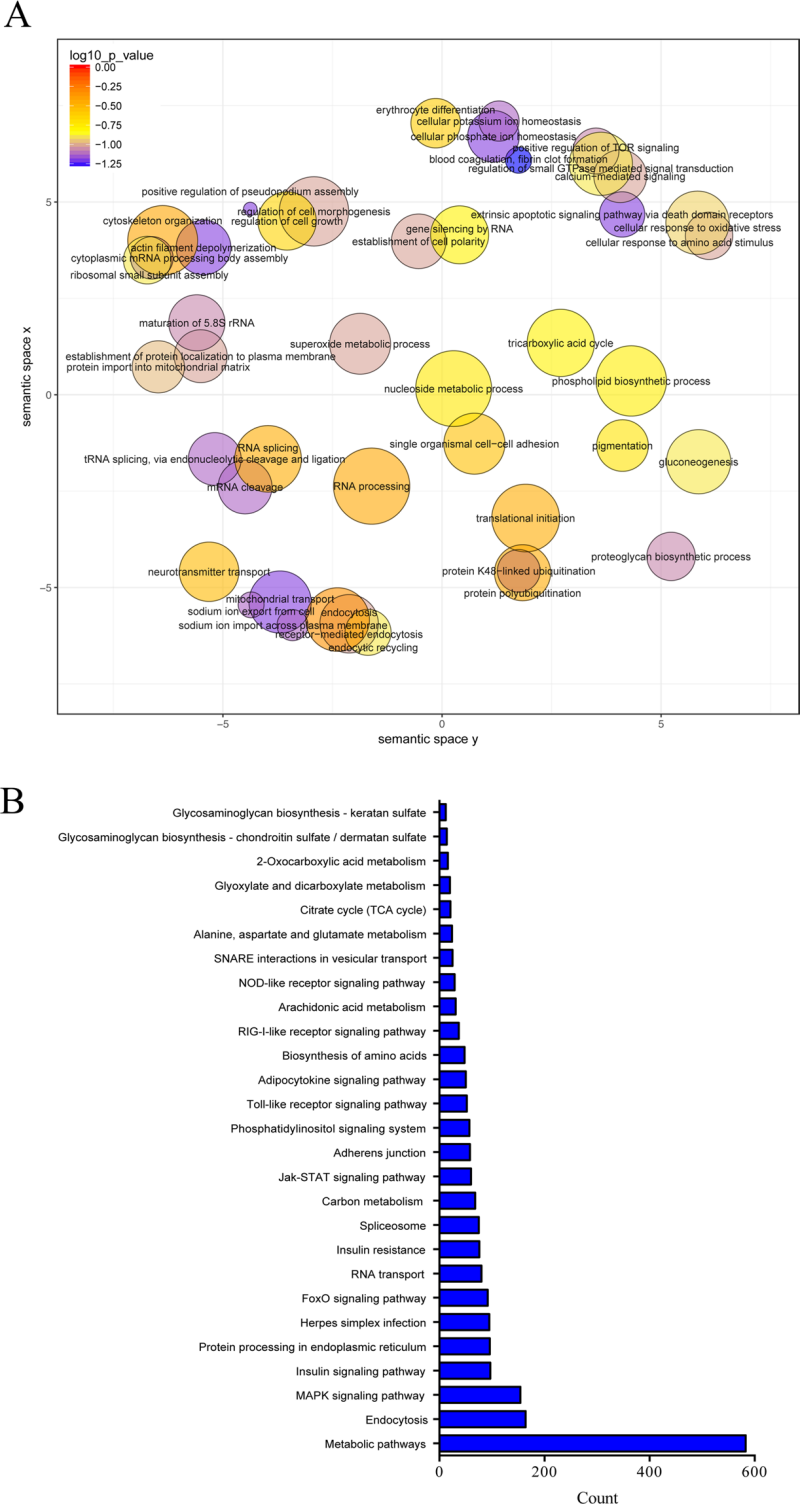
Annotation analysis of the neighboring genes of common lncRNAs modulated in WT and *rag1*^+/−^ zebrafish during viral infection. (**A**) GO enrichment analysis, (**B**) KEGG pathway analysis.

Furthermore, the neighboring genes of lncRNAs that were expressed in both WT and r*ag1*^+/−^ zebrafish were annotated by GO enrichment and KEGG analysis (Fig. 8A,B). In this case, the GO terms were mainly associated with metabolic processes and RNA processing, although, as expected, several terms directly related to the immune system were also observed, such as positive regulation of TOR signaling, extrinsic apoptotic signaling pathway via death domain receptors, blood coagulation-fibrin clot formation, superoxide metabolic process and cellular response to oxidative stress (Fig. 8A). Numerous terms associated with cellular shape and cytoskeleton organization were also potentially regulated by lncRNAs induced after SVCV infection, regardless of the zebrafish line (Fig. 8A). The KEGG analysis showed a large number of processes linked to viral infection, such as endocytosis, the MAPK signaling pathway, herpes simplex infection, the Toll-like receptor signaling pathway, the RIG-I-like receptor signaling pathway, and the NOD–like receptor signaling pathway (Fig. 8B).

### qPCR validation of differentially expressed lncRNAs

**Figure 9:**
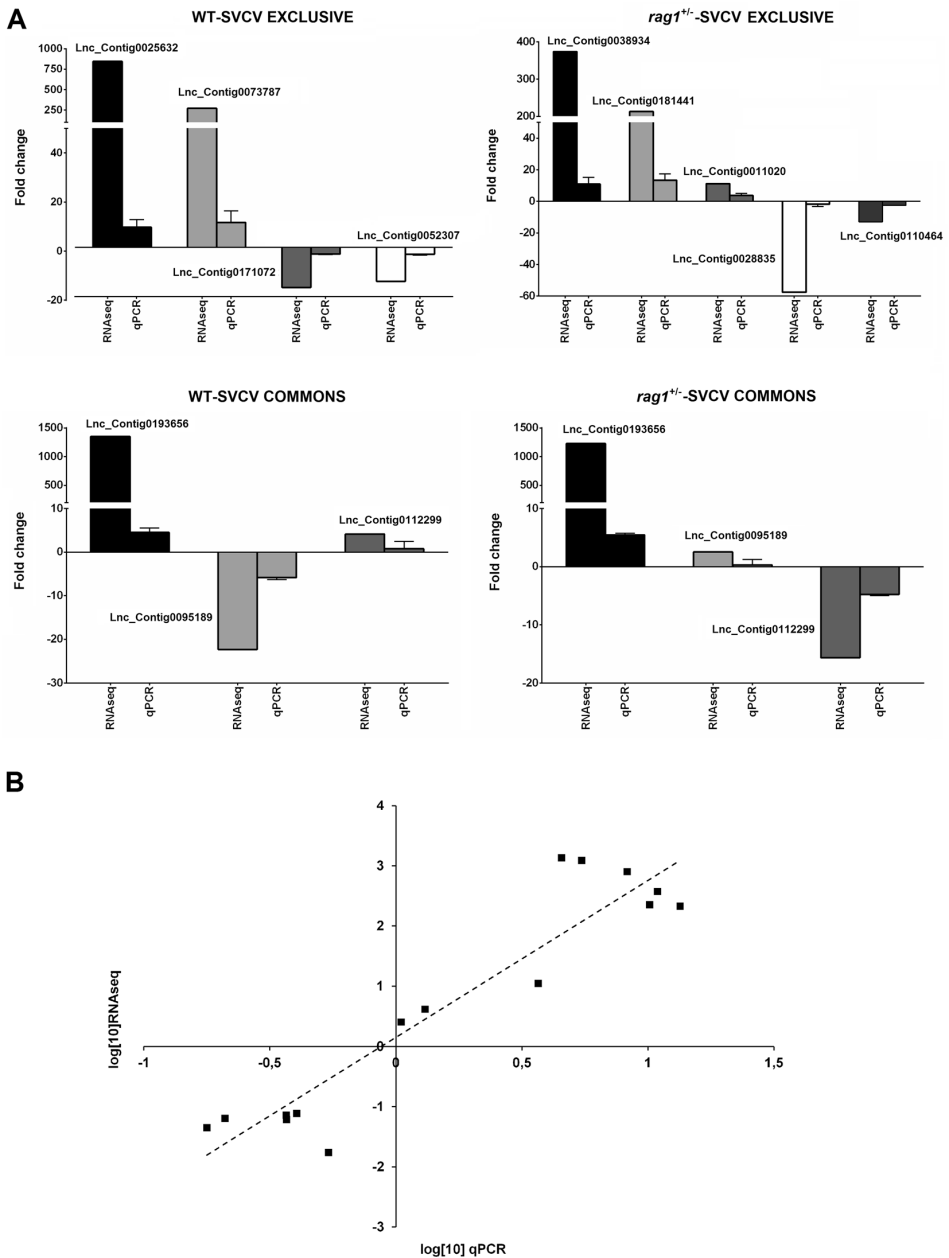
qPCR validation of the lncRNA expression. (**A**) qPCR and *in silico* fold-change values of twelve lncRNAs modulated after SVCV challenge in WT and/or *rag1*^+/−^ zebrafish transcriptomes, (**B**) Correlation between the qPCR and RNA-Seq fold-change values evaluated using Pearson’s correlation coefficient (r = 0.94).

Twelve putative lncRNAs were evaluated by qPCR. Among these lncRNAs, four were exclusively modulated in WT after SVCV infection, five were exclusively modulated in *rag1*^+/−^, and 3 were affected by the infection in both zebrafish lines. From the lncRNA qPCR results, similar expression profiles were observed between the fold-changes obtained via in silico analysis and the fold-changes determined by qPCR (Fig. 9A). Furthermore, a positive correlation was observed between the RNA-Seq and qPCR fold-change values evaluated using Pearson’s correlation coefficient (r = 0.94) (Fig. 9B).

## Discussion

Members of the Rhabdoviridae family are important pathogens of valuable fish species in world aquaculture^33–35^, with SVCV being the main pathogenic rhabdovirus of cyprinids, such as zebrafish^17^. Evidence of the participation of lncRNAs in the antiviral immune response in teleosts is scarce^9,11^, and there is a total lack of such evidence related to fish rhabdoviruses. In the last few years, lncRNAs that are differentially expressed in virus-infected cells have been linked to increases or decreases in the expression of typical antiviral genes. Furthermore, the described modulatory functions of these lncRNAs have mainly been related to their impact on the expression of interferon-stimulated genes (ISGs)^36–39^. Additionally, viruses can alter the expression of certain host lncRNAs to favour their own replication^40,41^.

Recently, modulation of lncRNAs was described after rhabdovirus infection in mice infected with the rabies virus (RABV)^42^. The authors identified a total of 140 lncRNAs that were differentially expressed at 8 days post-infection. Moreover, by using GO and KEGG pathway analyses, the authors identified genes associated with immune-related processes (e.g., apoptosis, Toll-like receptor, TNF, MAPK, B cell receptor and T cell receptor signaling pathway genes) as potential targets of these lncRNAs, suggesting putative participation of these lncRNAs in the immune response^42^. In this regard, putative roles of lncRNAs have been described in Atlantic salmon in response to different pathogens (infectious salmon anaemia –ISA– virus, the intracellular bacterium *Piscirickettsia salmonis* and the ectoparasite copepod *Caligus rogercresseyi*)^11^. Moreover, in Atlantic salmon, tissue-specific lncRNA expression has been reported in response to ISAV, and a correlation between the expression of specific lncRNAs and antiviral transcript expression has been suggested^9^.

LncRNAs participate in the modulation of innate and adaptive immune responses^2^. Since the majority of lncRNAs are expressed in specific cell types^2^, the use of mutant animals lacking a particular immune cell type could help to elucidate the specific roles of the different lncRNA sets. This is the case for the *rag1*^−/−^ zebrafish mutant, which is a T and B cell-deficient model, and as a consequence, these animals rely exclusively on their innate immunity^43^. It was previously reported that, under naïve conditions, adult homozygous *rag1*^−/−^ fish possess enhanced innate immunity to compensate for the absence of adaptive immunity, and these fish have been shown to be more resistant to SVCV compared to WT fish^31,32,44^. In this study, when we analyzed the survival rates of WT and heterozygous *rag1*^+/−^ mutants after challenge with SVCV, we did not observe significant differences in mortality. Therefore, because homozygous fish possess increased innate immunity, the use of heterozygous *rag1*^+/−^ mutants could improve the identification of lncRNAs associated with adaptive immunity due to the presence of B and T lymphocytes in these animals. We assume that after SVCV challenge, the expression of those genes involved in the production of immunoglobulins and TCR recombination should be increased in *rag1*^+/−^ fish compared to WT. In this work, we wanted to take advantage of this model to study lncRNA profiles after early infection with SVCV in both WT and *rag1*^+/−^ zebrafish.

A total of 12,165 putative lncRNA sequences were identified in zebrafish kidneys, 10,859 of which were common to the two lines, while 915 were excusive to WT, and 337 were only found in *rag1*^+/−^. Interestingly, when the functionality of the neighboring protein-coding genes was analyzed, the biological process category immune system process appeared to be represented in *rag1*^+/−^ but not in WT zebrafish. This difference could indicate that these exclusively expressed lncRNAs allow a compensatory response to the partial deficiency of functional Rag1 in *rag1*^+/−^ fish, increasing the expression of genes associated with acquired immunity. Indeed, from the lncRNA genome location, five lncRNAs were located near *rag1* and *rag2* genes, and two of them were up-regulated in *rag1*^+/−^ compared to WT fish. However, future functional studies are necessary to understand the role of these lncRNA. The other biological processes observed among the *rag1*^+/–^ exclusive lncRNAs were associated with the cellular response, cell signaling and communication terms. WT-exclusive lncRNAs were located close to genes that are mainly involved in metabolic processes. If we take into consideration the energy cost of the immune response^45^, the compensatory and/or deficient mechanisms present in the mutant fish could explain the differences in metabolism-related GO terms between the two lines, although we cannot discard other possible collateral effects of the mutation.

The enrichment analysis performed for the neighboring genes of lncRNAs that were exclusively modulated in *rag1*^+/−^ after SVCV challenge showed several immune-related GO terms, including T cell-mediated immunity, regulation of adaptive immune response, and regulation of adaptive immune response/built from immunoglobulin, among others. These processes are mediated by the adaptive immune system, which is nonexistent in *rag1*^−/−^ fish but presumably partially present in *rag1*^+/−^. This difference is probably due to a compensatory mechanism present in *rag1*^+/−^ zebrafish to effectively oppose SVCV infection with an efficient B and T lymphocyte response. Therefore, we can presuppose the involvement of these specific lncRNAs in the modulation of adaptive responses.

Furthermore, the GO enrichment analysis of the genes surrounding lncRNAs that were induced after viral infection in both WT and *rag1*^+/−^ showed high representation of terms related to RNA processing and metabolism. The high representation of RNA processing mechanisms (RNA processing, translational initiation, mRNA cleavage, RNA splicing, tRNA splicing, maturation of 5.8S rRNA, ribosomal small subunit assembly, and cytoplasmic mRNA processing body assembly) highlight the relevance of SVCV-induced lncRNAs in the transcriptional and translational machinery of the host cells. An increasing number of lncRNAs have been linked to the modulation of these processes, especially splicing regulation^46^. Among the identified metabolic processes, we found terms consistent with the activation of immune cells (tricarboxylic acid cycle, gluconeogenesis, superoxide metabolic process, and phospholipid biosynthetic process). Increased lipid biosynthesis and decreased fatty acid oxidation are also key mechanisms involved in such activation^47^. Indeed, the enriched term positive regulation of TOR signaling is also closely related to this process. The mammalian target of rapamycin (mTOR) is directly involved in the induction of aerobic glycolysis and promotes the synthesis of fatty acids^48^. mTOR is also an inhibitor of autophagy^49^, a controversial mechanism that has been described as both antiviral and pro-viral machinery^50^. The extrinsic apoptotic signaling pathway via death domain receptors, which was enriched in this analysis as well, is especially relevant in the elimination of virus-infected cells^51^. On the other hand, lncRNAs regulating blood coagulation-fibrin clot formation are also expected due to the highly haemorrhagic nature of SVCV.

Other interesting groups of enriched GO terms identified after infection were related to cell morphogenesis (actin filament depolymerization, cytoskeleton organization, positive regulation of pseudopodium assembly, regulation of cell morphogenesis). Virion binding to the cell surface triggers signaling mechanisms that alter the cell surface and activate endocytosis^52^. One of the most enriched KEGG pathways among the potential targets of the SVCV-induced lncRNAs in both lines was endocytosis. LncRNAs that are potentially involved in virion internalization remain unexplored, but some lncRNAs participating in cytoskeletal remodeling have been identified in tumors^53,54^.

The KEGG pathway analysis of SVCV-induced lncRNAs indicated that these lncRNAs were mainly neighbors of genes involved in metabolic pathways and, particularly, that the insulin pathway showed strong representation. It has been well established, especially for the hepatitis C virus, that infection induces insulin resistance^55,56^. Numerous lncRNAs have been linked to insulin resistance and diabetes, and these lncRNAs therefore represent a very interesting target for novel therapeutic strategies^57^. Nevertheless, this effect could be related to changes in glucose metabolism suffered by the immune cells upon activation. As expected, numerous immune-related pathways were also identified (MAPK signaling pathway, herpes simplex infection, Toll-like receptor signaling pathway, RIG-I-like receptor signaling pathway and NOD-like receptor signaling pathway), including some common pathways similar to those observed in mice after RABV infection^42^.

Therefore, we can conclude that SVCV infection in zebrafish induces strong modulation of lncRNAs with a potential role in the immune response, and the heterozygous *rag1* mutant could be a useful tool for the identification of lncRNAs linked to adaptive immunity. Furthermore, the lncRNAs that were modulated in both lines after viral infection represent an excellent source of information for further functional studies focused on the identification of their specific roles under infection. Nevertheless, we are far from understanding all of these coding gene-lncRNA interactions in detail. Future functional investigations could clarify the specific roles of the various lncRNAs modulated in response to the virus.

## Supplementary information

Supplementary Figures 1-2

Supplementary Table S1

Supplementary Table S2

Supplementary Table S3

Supplementary Table S4

## Data Availability

The raw read sequences were deposited in the Sequence Read Archive (SRA) (https://www.ncbi.nlm.nih.gov/sra) under accession number PRJNA532380.
