# Supplementary material for: Comparative modulation of lncRNAs in wild-type and rag1-heterozygous mutant zebrafish exposed to immune challenge with spring viraemia of carp virus (SVCV)
Source: Sci Rep. 2019 Oct 2;9:14174. doi: 10.1038/s41598-019-50766-0 (PMC6775065; doi:10.1038/s41598-019-50766-0)

Comparative modulation of lncRNAs in wild-type and *rag1*-heterozygous mutant zebrafish exposed to immune challenge with spring viraemia of carp virus (SVCV)

Valentina Valenzuela-Muñoz[1*], Patricia Pereiro[1,2*], Margarita Álvarez-Rodríguez[2], Cristian Gallardo-Escárate[1], Antonio Figueras[2], Beatriz Novoa[2]

[1]Laboratory of Biotechnology and Aquatic Genomics, Interdisciplinary Center for Aquaculture Research (INCAR), University of Concepción, Concepción, Chile.

[2]Instituto de Investigaciones Marinas (IIM), CSIC, Vigo, Spain.

*: these authors have contributed equally to this work

Corresponding Authors

Beatriz Novoa, *Instituto de Investigaciones Marinas (IIM), CSIC, Eduardo Cabello 6, 36208, Vigo, Spain (beatriznovoa@iim.csic.es*)

C Gallardo-Escárate*, Universidad de Concepción, P.O. Box 160-C, Concepción 4030000, Chile (e-mail: crisgallardo@udec.cl)*

Supplementary figures

Figure S1. Survival curve of WT and *rag1*$^{+/-}$ zebrafish infected with SVCV.

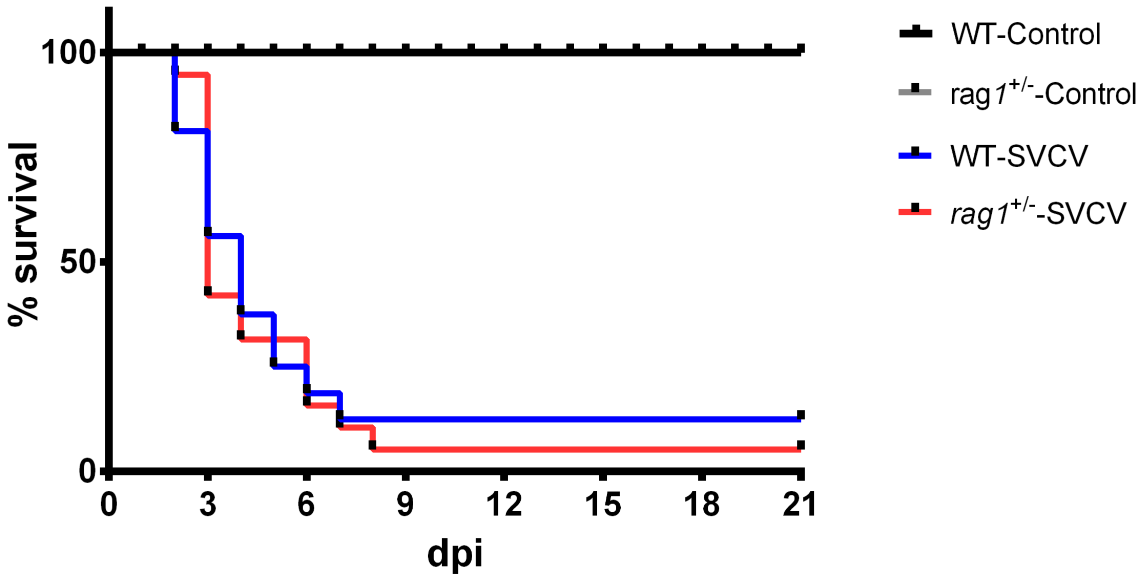

Figure S2. GO enrichment analysis of exclusive coding RNAs differently expressed in WT and *rag1*$^{+/-}$ zebrafish during SVCV infection.

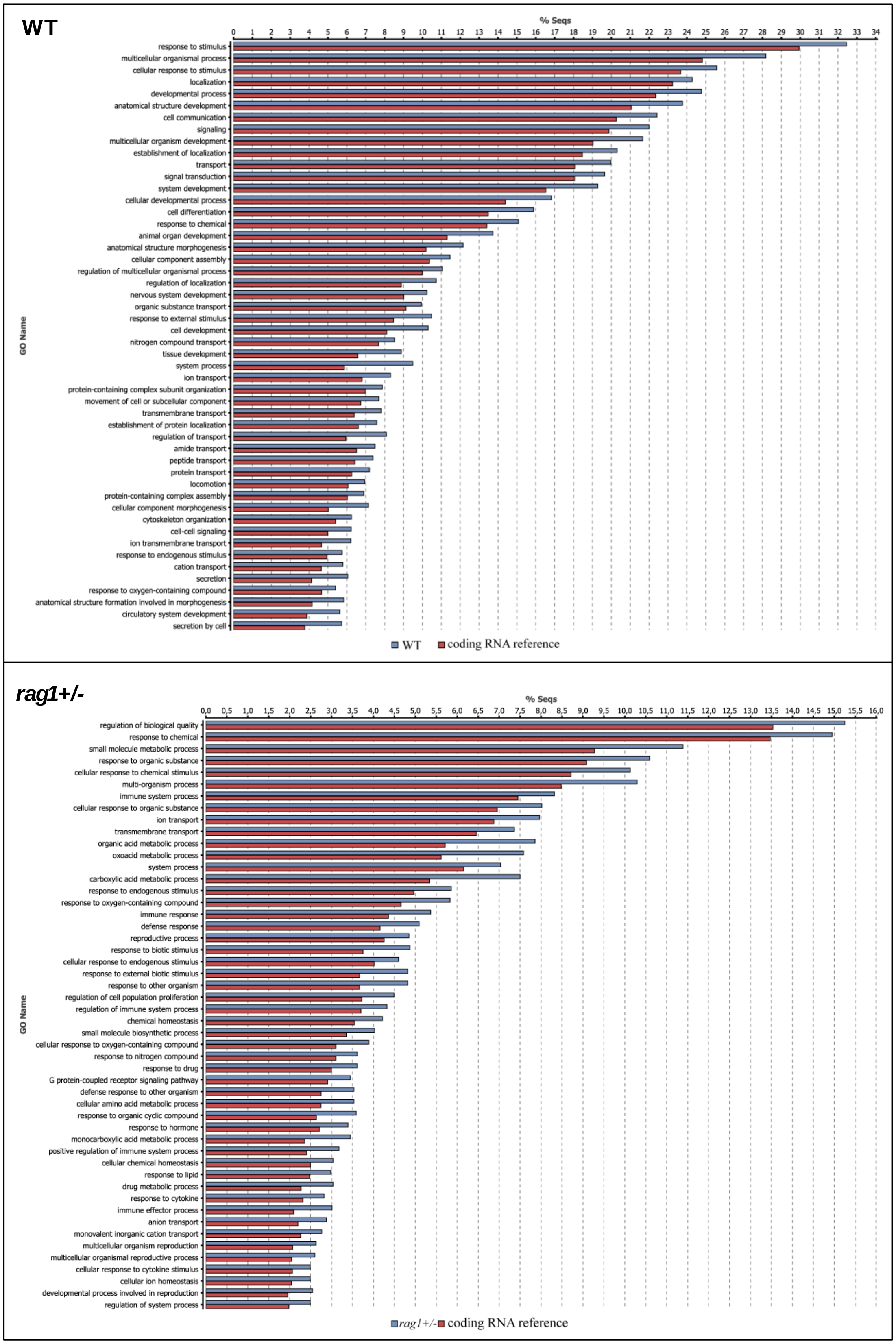

Supplement: Supplementary file 1 — Supplementary Figures 1-2 [file 41598_2019_50766_MOESM1_ESM.pdf]
